# Titanium Dioxide Nanoparticle-Based Interdigitated Electrodes: A Novel Current to Voltage DNA Biosensor Recognizes *E*. *coli* O157:H7

**DOI:** 10.1371/journal.pone.0139766

**Published:** 2015-10-07

**Authors:** Sh. Nadzirah, N. Azizah, Uda Hashim, Subash C. B. Gopinath, Mohd Kashif

**Affiliations:** 1 Institute of Nano Electronic Engineering,Universiti Malaysia Perlis, 01000 Kangar, Perlis, Malaysia; 2 Department of Electrical and Electronic Engineering, Faculty of Engineering, Universiti Malaysia Sarawak, 94300 Kota Samarahan, Sarawak, Malaysia; Argonne National Laboratory, UNITED STATES

## Abstract

Nanoparticle-mediated bio-sensing promoted the development of novel sensors in the front of medical diagnosis. In the present study, we have generated and examined the potential of titanium dioxide (TiO_2_) crystalline nanoparticles with aluminium interdigitated electrode biosensor to specifically detect single-stranded *E*.*coli* O157:H7 DNA. The performance of this novel DNA biosensor was measured the electrical current response using a picoammeter. The sensor surface was chemically functionalized with (3-aminopropyl) triethoxysilane (APTES) to provide contact between the organic and inorganic surfaces of a single-stranded DNA probe and TiO_2_ nanoparticles while maintaining the sensing system’s physical characteristics. The complement of the target DNA of *E*. *coli* O157:H7 to the carboxylate-probe DNA could be translated into electrical signals and confirmed by the increased conductivity in the current-to-voltage curves. The specificity experiments indicate that the biosensor can discriminate between the complementary sequences from the base-mismatched and the non-complementary sequences. After duplex formation, the complementary target sequence can be quantified over a wide range with a detection limit of 1.0 x 10^-13^M. With target DNA from the lysed *E*. *coli* O157:H7, we could attain similar sensitivity. Stability of DNA immobilized surface was calculated with the relative standard deviation (4.6%), displayed the retaining with 99% of its original response current until 6 months. This high-performance interdigitated DNA biosensor with high sensitivity, stability and non-fouling on a novel sensing platform is suitable for a wide range of biomolecular interactive analyses.

## Introduction

*Escherichia coli* (*E*. *coli*) O157:H7 was first discovered in1982 [1] and was considered the most virulent foodborne pathogenic bacteria in 1996 [2]. This type of *E*. *coli*is classified as anEnterohemorrhagic *Escherichia coli* (EHEC)[3], which can produce a Shiga-like toxin and cause life-threatening gastrointestinal infections, such as bloody diarrhoea[1], haemorrhagic colitis, renal failure and meningitis[4]. Many outbreaks, cases, and deaths associated with the bacteria have occurred with increasing frequency all over the world. It is estimated that more than 73,000 cases of EHEC-related illnesses and 61 deaths occur annually in the United States. The US Department of Agriculture’s Economic Research Services [5]reported that the medical costs, productivity losses, and loss of life from diseases caused by major food-borne pathogens total $6.9 billion per year[1]. Therefore, high specificity and sensitivity biosensors, which are a rapid and inexpensive method for the detection of *E*. *coli* O157:H7,are urgently needed.

Classical methods for the detection of pathogenic bacteria involve the following basic steps: pre-enrichment, selective enrichment, selective plating, biochemical screening and serological confirmation [6, 7]. Hence, the classical methods,such as culture and colony counting, gel electrophoresis, membrane blots and immunology-based methods,for detection of bacteria are time-consuming and tedious[1, 4, 8]. The results of such tests are often not available on the time scale desired in the clinical laboratory, which is why the development of alternative detection and identification technologies has become increasingly important in recent years. Researchers have developed a few methods based on various measuring principles such as a chemiluminescence assay[9, 10] and fluorescence[9]. However, these methods are label-based and qualitative measurements that use various chemicals to get results. In order to achieve the desired sensitivity, specificity and detection limit, a DNA-based pathogen detection method that requires an amplification method such as polymerase chain reaction (PCR)[10] was developed. However, PCR-based methods require expensive equipment, skilled personnel and labour-intensive gel-based detection that shows poor sensitivity and specificity [11]. PCR is not sensitive enough because it requires DNA sequence amplificationwith several rounds to provide a result.

In recent years, many attempts have been made to improve the sensitivity in pathogenic bacteria detectionthrough the use of biosensors[12]. Biosensor technologies play an important role in the detection of pathogenic bacteria because of their great potential to satisfy the in-field testing need for rapid, portable and low-cost detection. Until now, pathogen biosensors mainly included immunosensing,which is based on specific antibody-antigeninteractions[1, 3, 7]and DNA-based detection [13–16]. However, the instability of antibodies in harsh environments and the single-use nature of most immunosensors limit the practical application of these immunosensing-based biosensors. The field has turned to high-specificity DNA-based biosensors. There are three parts of DNA biosensors: 1. a solid surface of a transducer, 2. a single strand of DNA immobilized onto the surface (probe) and 3. sequence-specific single-stranded DNA (target) as the test sample[17]. The core of a DNA biosensor is based on the detection of the single-stranded target DNA by utilizing their hybridization with complementary probe sequences[18, 19]. Electrochemicaltransducers have been widely studied due to their unique advantages in the detection of DNA hybridization and have garnered considerable interest[13, 15, 20, 21]. However, electrochemical transducersrequiredifferent chemicals for the silanization, immobilization and hybridization processes, which create noise.In addition, the use of alternating current makes it unsuitable for portable devices. The primary reason to use DC is to make the sensor to be used for bedside analyses. For field or bedside analysis, battery is mostly preferred and it supplies DC. In addition, DC has more constant flow than AC, ultimately no variation in the voltage, will give more accurate and reproducible results.

Hence, we introduce a direct current (DC) picoammeter that provides simple, real-time and label-free detection. This sensitive DC picoammeter device could be integrated into existing detection schemes; the picoammeter detection is desirable to realize miniaturization and portability. Moreover, the low cost, minimal power requirements, and independence from optics make it an excellent candidate for DNA diagnostics. In addition, the picoammeter provides innovative routes for interfacing the nucleic acid recognition system with signal generation, and no activation chemical is needed to read small current changes during DNA reaction.

TiO_2_was used as the substrate on the sensing surface, because TiO_2_-basedsemiconductor metal-oxide material has attracted substantial interest due to their low cost and production flexibility. The biocompatibility of TiO_2_ nanoparticles in the development of biosensor promises better sensing performance and enhancing the electron-transfer kinetics. To increase the surface-to-volume ratio and enhance sensitivity, enabling the semiconducting conductance to be easily modulated by the target DNA for metal-oxide nanostructures have been proposed. The electrical characteristics of nanostructures with high surface-to-volume ratios can be easily modified using structure shape and geometry, which likely allows various degrees of depletion from the charge carriers when biomolecules are exposed to. The nanostructure increase the surface-to-volume ratios that gives higher surface reaction activity and strong adsorption of biomolecules capability [22].

There are three phases of TiO_2_ namely, brookite, anatase and rutile. Rutile structure has the most stable phase; while anatase and brookite are less stable than rutile. These three different phases have different energy gap. Among them, rutile has slightly lower energy gap compared to anatase [23–25]. Thus, rutile has better conductivity than anatasee. However, in order to get complete rutile phase, it needs extremely high temperature (~900°C) [26, 27]. This temperature has exceed the melting point of aluminium metal on interdigitated electrodes and it might damage the sensor. Therefore, mixing of both anatase and rutile structures in a TiO_2_ thin film can be considered as this sensor performs high stability. On the TiO_2_, we analysed the DNA duplex formation using the sequences from *E*. *coli* O157:H7, for which the target DNA tested was from both commercial source and lysed *E*. *coli* O157:H7.

## Materials and Methods

### Chemicals and Reagents

Titanium isopropoxide (Ti[OCH(CH_3_)_2_]_4_, 97%), acetic acid (AA), ammonium hydroxide [NH_4_OH] (27%), ethanol(C_2_H_5_OH), aluminium, buffered oxide etchant (BOE) solution, acetone, RD–6 and (3-Aminopropyl)triethoxysilane (APTES) were obtained from Sigma Aldrich, USA. Negative resist NR5-8000 was purchased from Futurrex Productivity Tools. All of the other chemicals were analytical reagent grade and purchased commercially. Deionized distilled water (DDI-water) was used throughout this experiment. The 30-base synthetic oligonucleotides were purchased from AITbiotech Pte. Ltd. (Singapore).

### Instruments

The crystallographic structures and phases were performed using PANalytical X-Pert Pro X-Ray Diffraction (XRD) using Ni-filtered CuKα radiation at room temperature. The morphologies of the TiO_2_ thin films were characterized by HITACHI SU8020 Field Emission Scanning Electron Microscopy (FESEM). The measurements for current to voltage were carried out by a 6487 Picoammeter/Voltage Source (Keithley).

### Fabrication Process of Interdigitated Electrodes (IDEs)

**Fig 1:**
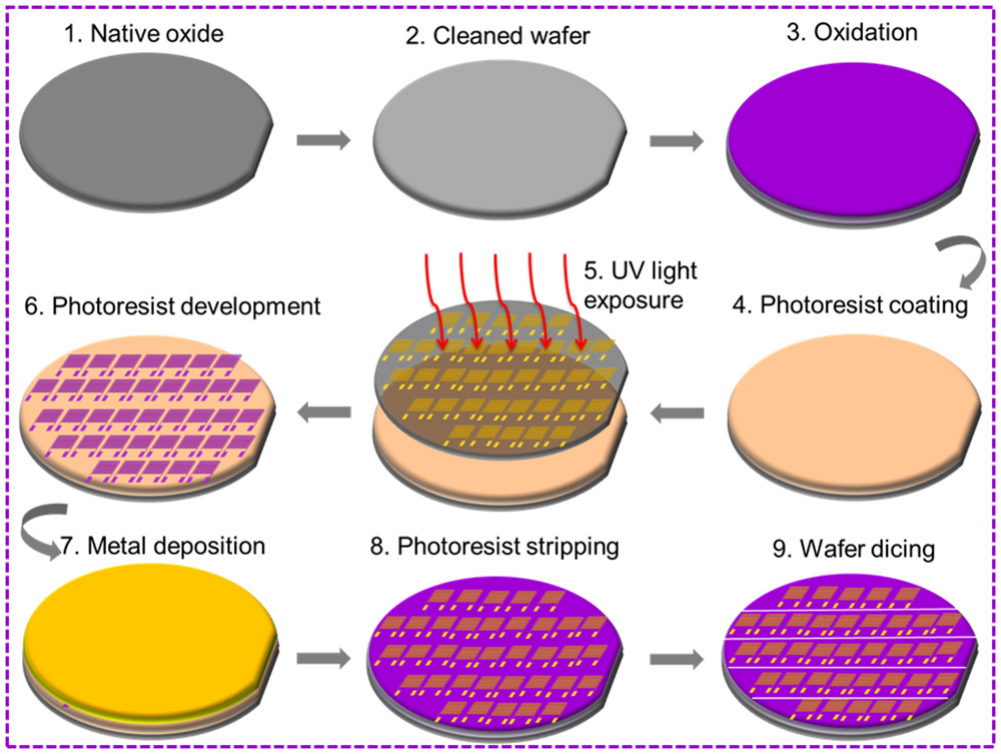
Interdigitated electrode fabrication process. Nine steps were followed to complete the fabrication of this device under room temperature.

A 4-inch p-type Si wafer was used as a substrate. There were six processes used to fabricate the IDEsas shown in Fig 1, including wafer cleaning, oxide layer growth, negative photoresist spin-coating, photoresist patterning, aluminium (Al) metal deposition, photoresist removing and wafer dicing. After cleaning the wafer from native oxide using buffered oxide etchant (BOE) and deionized water (DI-water), an insulation layer of SiO_2_ with a thickness of 310 nm at 1000°C was grown by wet oxidation. The oxide layer was used to isolate the deposited TiO_2_ film from silicon substrate in order to neglect the influence of current flow from silicon substrate. On top of that, a negative photoresist ma-N 1405was deposited by spin-coater with a spin speed of 2000 rpm. The resist was developed by RD–6 after exposure to UV-light for 240 s. Then, 240 nmAl metal was deposited via a thermal evaporator (Edwards Auto 306) at 3.0E-5 Torr. The photoresist was stripped using acetone until solid IDEs were seen, and it was diced to create single IDEs.

### TiO_2_ Solution Preparation

After Al IDEs were fabricated, they were coated with TiO_2_ solution on top of the fingers area only. The TiO_2_solution was prepared using TIP as a precursor. First, TIP was mixed with ethanol and stirred vigorously for 5 minutes. Then, 0.1 ml acetic acid was added drop wise as a stabilizer under stirring conditions and was heated on an 85°C hot plate. The molar ratio of mixture (TIP: ethanol: acetic acid) was 1:9:0.1[28]. The measured value of pH of the solution was3.8 at 25°C. After mixing for three hours, a clear solution was obtained. After aging for 24 hours, the solution was deposited on silicon dioxide (SiO_2_) substrates by a spin-coating technique with a speed of 2000 rpm. Following each coated layer, the films were dried at 175°C for 15 minutes and annealed at 450°C for 1 h. The TiO_2_thin film had sufficient thickness after 3 layers of coating. The chemical structure of the films was examined by XRD,and the surface morphology of the films was observed by FESEM.

### Silanization of TiO_2_ Nanoparticles by APTES

TiO_2_ nanoparticles were functionalized with APTES using a silanization process covering the surface by self-assembly. The film was silanized with 0.02 M APTESand it was prepared inside a fume hood. As much as 2.5 μl APTES was dropped on the TiO_2_ nanoparticle-based transducer surface to become an ‘active’ layer and left to dry in dry cabinet for 3h. Then, the surface was rinsed with deionized (DI) water. No activation chemical is required because of the ability of the picoammeter to read extremely small current changes during the immobilization and hybridization process.

### Immobilization and Hybridization DNA on TiO_2_ Nanoparticle-Based Transducer

Carboxyl-modified synthetic probes and target oligonucleotides were purchased specific to *E*. *coli* O157:H7. The 30-base synthetic oligonucleotides with base sequences are as follows: 30-mer probe: (5’-(COOH) AAC GCC GAT ACC ATT ACT TAT ACC GCG ACG–3’), 30-mer complementary: (5’-CGT CGC GGT ATA AGT AAT GGT ATC GGC GTT–3’), 30-mer non-complementary: (5’-GCA GCG CCA TAT TCA TTA CCA TAG CCG CAA–3’), and the single base mismatch oligonucleotide (5’-CGT CGC GGT ATA ACT AAT GGT ATC GGC GTT–3’) for the specificity test of the probe. Stock solutions of all oligonucleotides were prepared in autoclaved ultrapure water (> 18MΩ) to obtain a 10 μM solution and kept frozen (-20°C). Similarly, DNA from the lysed *E*. *coli* O157:H7 was tested.

**Fig 2:**
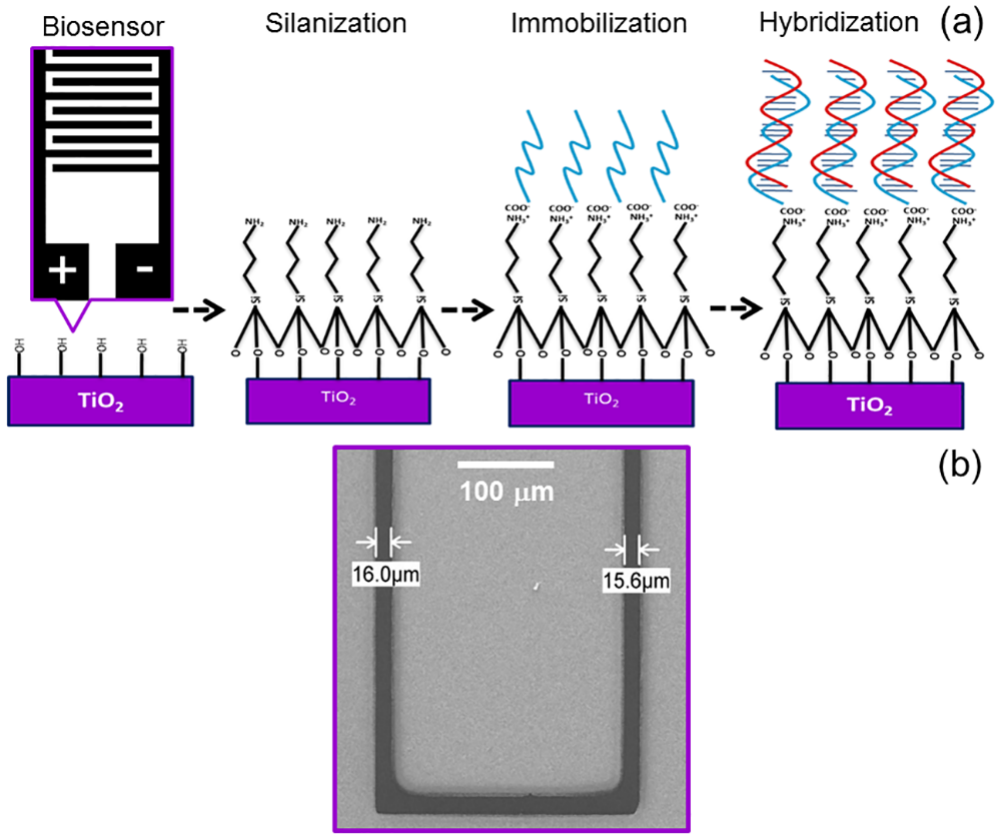
Preparation of sensing surface. (a) Schematics of the DNA immobilization and hybridization process. This resistive DNA biosensor with titanium dioxide nanoparticles enhances the current signal that eliminates PCR. (b) SEM image of interdigitated electrodes. The average gap size between two aluminum finger-shaped electrodes is 16 μm.

After the modification of the TiO_2_ nanoparticle-based transducer with APTES, 2.5μl of probe DNA was immobilized on the transducer surface to form a recognition layer by the covalent amide bond between the carboxyl group, which already modified the DNA probe sequences, and the amine group at APTES. The transducer surface was rinsed with DI water to remove unbound probe DNA. Finally, the hybridization reaction was performed with the complementary DNA by dropping 2.5μl. Then, the surface was rinsed with DI water before taking an electrical measurement. Current measurements were carried out before the probe DNA immobilization, after the probe DNA immobilization, and after the hybridization processes to determine the conductivity changes after each process (Fig 2a).

### Field emission scanning electron microscopy (FESEM) Measurements

FESEM (HITACHI SU8020) was used to study morphological structure of TiO_2_ thin film. It was scanned under room temperature and operating at 15kV with 100k magnification. Sample was carefully placed at the sample holder using tweezer and the height between lens and the sample was adjusted. When it is ready to be scanned, voltage was applied and electron was generated for scanning purpose. Finally, an image was come out on the PC screen and the structure was measured directly using the software.

### X-ray Diffraction (XRD) analyses

The TiO_2_ film that already coated on the SiO_2_ substrate was stick on the plate and it was inserted into a holder and ready to be scanned. The structural properties of TiO_2_ thin film was investigated using PANalytical X’Pert Pro X-ray diffractometer with Cu Kα radiation at λ = 1.5406 Å under room temperature. The X-ray diffraction (XRD) pattern was recorded in the range of 20° to 60° operating at 45 kV and 20 mA.

## Results and Discussion

Metal nanoparticle mediated sensing is a potential way to generate high-performance sensors to recognize a wide range of biomolecules from smaller ions to whole cells. Among different nanoparticles proposed, metal oxide nanoparticlefacilitates redox reaction upon interactmolecules lead to change in the electrical conductivity [29]. Moreover, higher numbers of oxygen groups, in metal oxide nanoparticles, promote the sensitivity of the biosensors. Recently, there are tremendous attentions of using oxide material for sensor fabrication purpose. The oxide material should be a functional biocompatible and non-toxic material. Besides that, the surface must has the ability to absorb strongly and has the capability to provide suitable microenvironments for the immobilization of biomolecules [22]. The most crucial thing, with the use of oxide material, it can improve biosensing characteristics by enhancing the electron transfer between the device and the biomolecules. Titanium dioxide (TiO_2_) is one of the metal oxides, widely attracted for the sensing applications, due to its physical strength, high chemical resistance, good electrical conductivity and an ideal oxidizing agent [30]. TiO_2_ has been selected to be fabricated as a DNA Biosensor due to its high chemical and temperature stability [31–33]. With the aim to commercialize our sensor, we chose TiO_2_ to sustain for a long period of time.Further, metal nanoparticle assisted bacterial detections attested for a wide range of medical diagnosis [12]. In the present study, we have generated a novel interdigitated electrodes (IDEs) DNA biosensor, which could recognize pathogenic *E*. *coli* O157:H7 by picoammeter measurements and displayed higher sensitivity and specificity.

### Characterization of the Nanoparticle Device

A DNA biosensor was fabricated by the combination of common IDEs with nanotechnology-synthesized TiO_2_ using a sol-gel method. To achieve an accurate and sharp IDE design, a lift-off process is preferred during pholithotography. This technique allows precise control of the IDE design; the gap size between the electrodes can be controlled more accurately before metal is deposited on it. However, in the lift-off process, there are challenging issues during the development process of this negative photoresist. The patterned photoresist may remain at the edges after lift-off. These ears protrude from the patterned photoresist and may cause electrical short circuit according to the connection with the opposite electrode.

In this study, we control two steps in order to prevent ear formation after fabrication. It is important to horizontally immerse the substrate into the RD6 during the development process and keep it stationery until this process is completed. Strictly no movement is allowed; otherwise, it creates a wave-like electrode edge. Fig 2b shows the SEM image of aluminium IDEs with a 16-μm gap without any ear-effect remaining. Another crucial step is during strip resist, which is the final step after aluminium metal deposition. The substrate is immersed carefully as mentioned above until the resist is completely stripped to prevent excessive metal from stickingin the small gap between the fabricated electrodes.

Next, TiO_2_ solution was prepared in an acidic solution to achieve with good electrical properties. Many researchers refuse to fabricate TiO_2_for a device because ofthe difficulty of the synthesization process and its high chemical stability, especially after passing the annealing process. Only strong acid is able to remove TiO_2_. Thus, a thin masking layer of silver metal was deposited on the fabricated contact pads before the TiO_2_ solution was deposited on it. At this point, no TiO_2_was deposited onto the aluminium pads, and therefore, it did not affect the output current of this device because TiO_2_ nanoparticles control the quantized electron flow.

**Fig 3:**
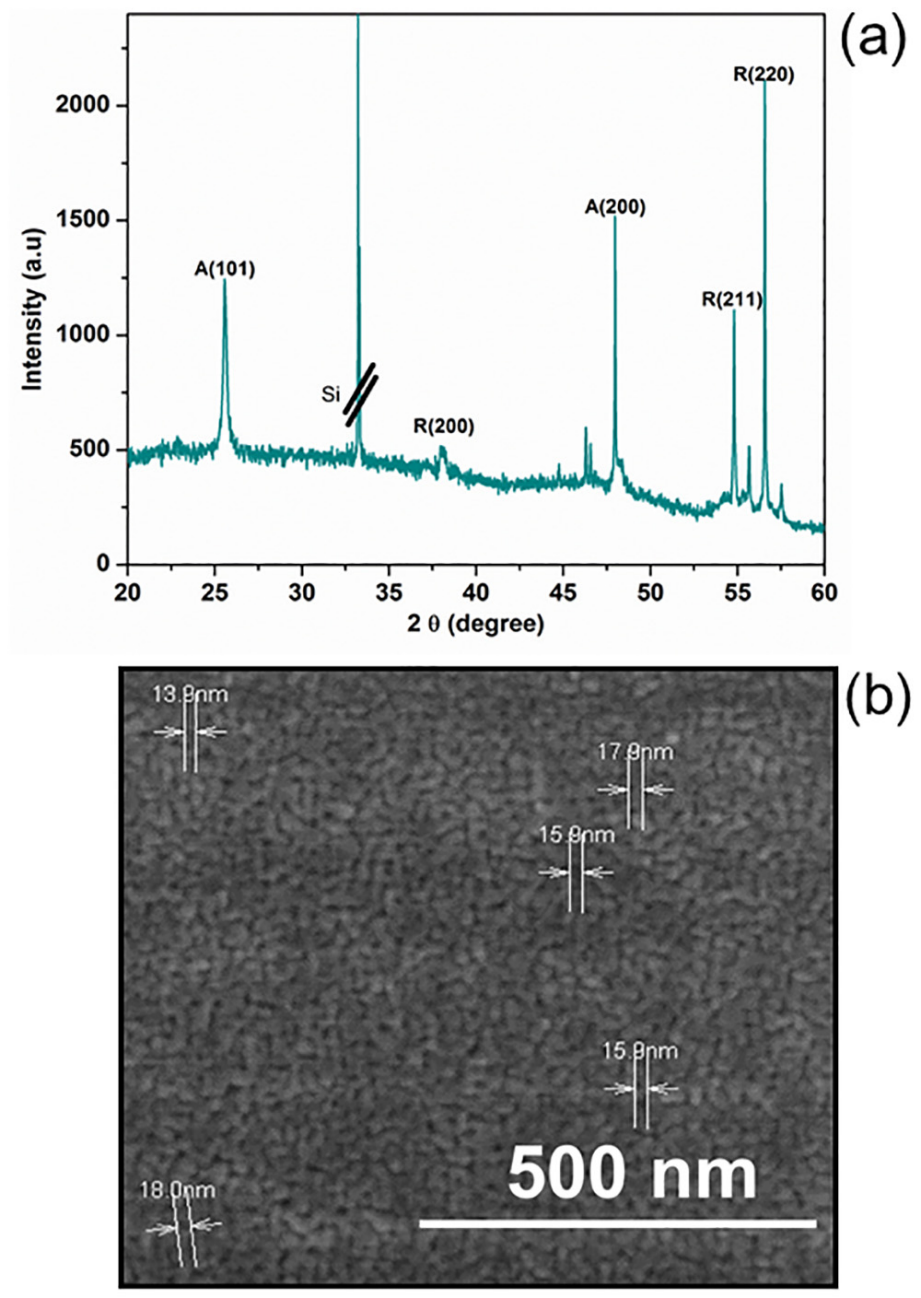
Measurements on sensing surface. (a) XRD spectra of TiO_2_ thin film. Annealed TiO_2_ thin film was characterized to get highsurface crystallinity. (b) FESEM image of TiO_2_ nanoparticles. 100 k magnification was used to scan the TiO_2_ structure under ambient temperature.

The deposited TiO_2_ film should be crystalline with high surface roughness for biosensor application. The crystal structure of the TiO_2_ was analysed by X-ray diffraction (XRD). Fig 3a displays the XRD spectra of TiO_2_ thin film annealed at 450°C for 1 h. There are anatase (A) and rutile (R) structures at peaks (101), (200) and (200), (211), (220), respectively. The XRD spectra revealed that all of the deposited films were polycrystalline in nature with tetragonal crystal structure. The values obtained agreed well with the standard peak values of XRD pattern of TiO_2_ (Joint Committee on Powder Diffraction Standard, JCPDS data cards (21–1272 and 21–1276),[28].

The average crystalline size can be calculated using the Scherer equation

r=0.9λ/Bcosθ (1)

where λ is the X-ray radiation, *2Ɵ* is the diffraction angleand B is the full-width at half-maximum of the diffraction peak.

The high intensity of the peaks shows high degree of TiO_2_ periodicity (not agglomerated). The calculated crystallite size for TiO_2_ is 18 nm. It can be proved through the FESEM image.

The morphological structures of TiO_2_ particles were analysed using FESEM (Fig 3b). The particle diameter was determined by measuring the long axis of each particle. The particle growth was uniform, and no agglomeration occurred. The average particle size was ~16 ±2nm, which is in agreement with the XRD result.

**Fig 4:**
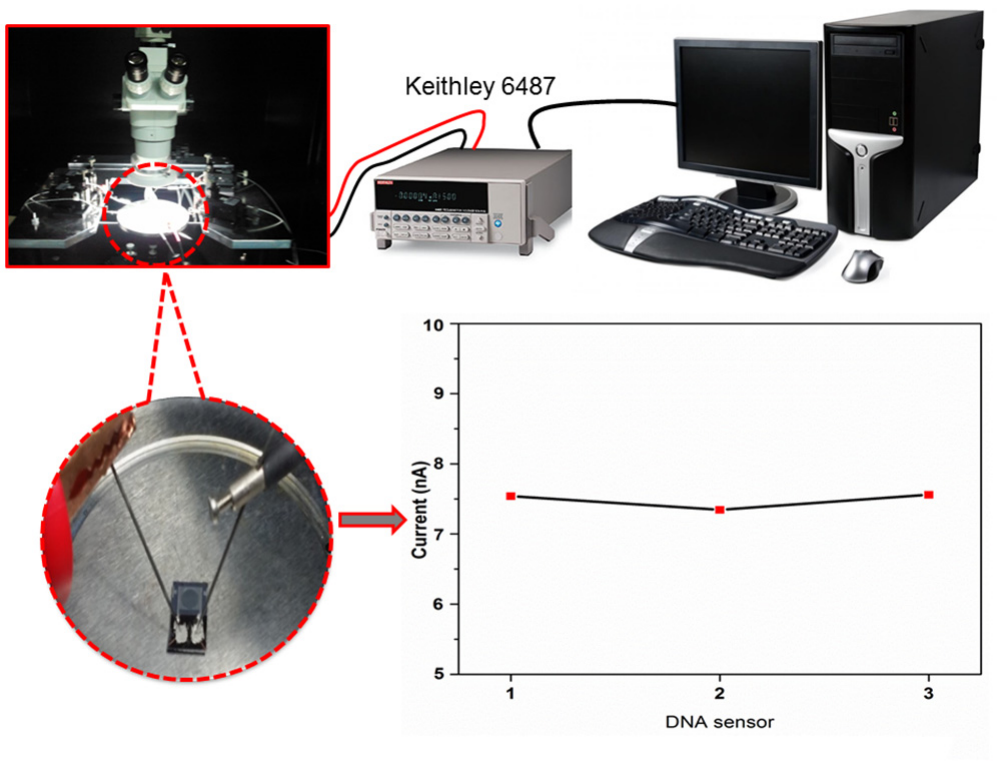
Experimental set-up of picoammeter for the TiO_2_ nanoparticle-based DNA biosensor. Current responses show ability of the device to retain the same output current after hybridization and dehybridization of 1 μM targeted DNA.

**Fig 5:**
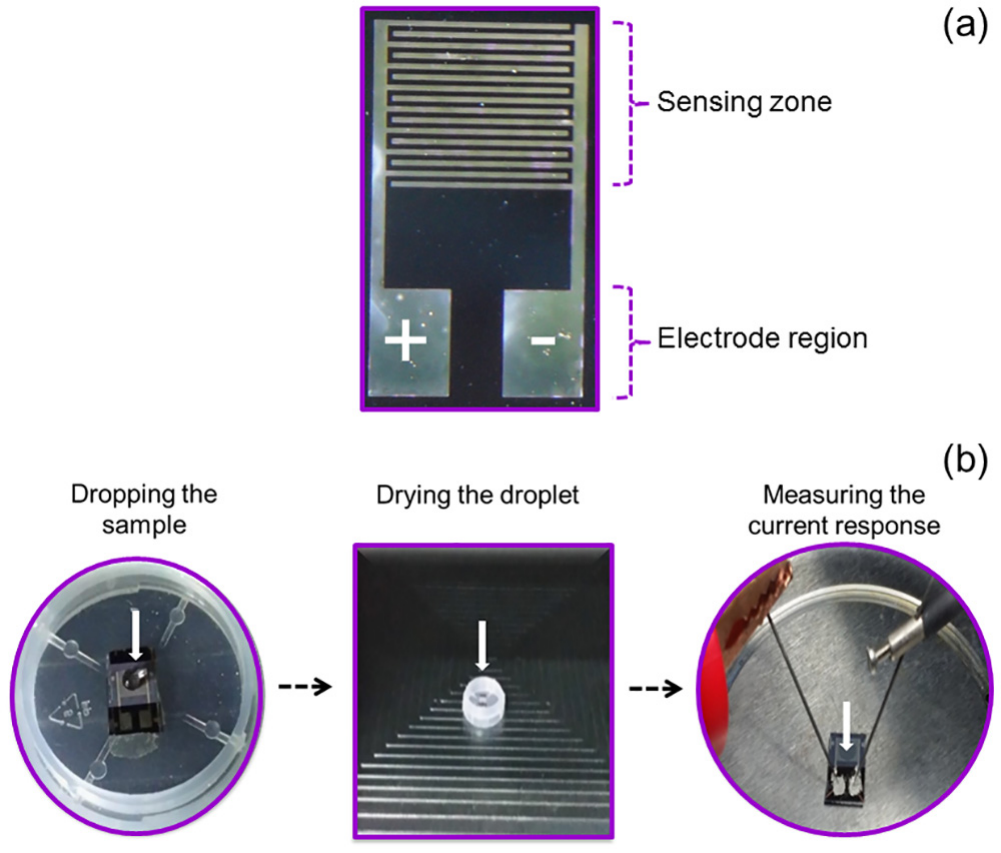
Steps were used for picoammeter-TiO_2_ nanoparticle DNA biosensor. (a) Sensing surface. The device has sensing zone that uniformly covered with TiO_2_ nanoparticles on top of the fabricated aluminum electrodes. (b) Three steps involved in the measurements. It includes dropping of sample, drying the droplet and measuring the current response.

In order to monitor the device performance,the extremely small electrical current of the TiO_2_ nanoparticles was measuredby a picoammeter in the small voltage range from 0V to 1V. This was instead of using a conventional electrochemical method, which is tedious due to the use of various chemicals and high noise. The ion migration of the *E*. *coli* O157:H7 DNA probes and target towards the synthesized TiO_2_ nanoparticles can be monitored by this picoammeter. The experimental setup of the picoammeter is visible in Fig 4. Finally, additional simple steps for *E*. *coli* O157:H7 DNA sample detection can be seen in Fig 5a and 5b. Only three steps were implemented: drop, dry and measure for the self-assembly, immobilization, and hybridization processes, respectively. The basic measurements have shown consistency with the DNA immobilization (Fig 4).

### Immobilization of the Synthesized *E*. *coli* O157:H7 Single-Stranded Probes

A prerequisite for every possible application is the proper surface functionalization of TiO_2_ nanoparticles, which determines their interaction with the environment [34]. The immobilization process is the most crucial step in fabricating a DNA sensor. A single-strandedDNA (ssDNA) *E*. *coli* O157:H7 probe modified with a carboxyl group was introduced on the TiO_2_ nanoparticle surface inorder to detect the presence of the target nucleotide sequences that were complimentary to the probe. For commercialization purposes, a DNA biosensor is developed until the immobilization step, and users need only single-stranded target DNA to be complementaryto the binding probe. Thus, it is important to know the lifetime of the oligomer probe on the fabricated DNA biosensor before it goesto commercialization. To test the stability of this sensor, 10 μM carboxyl-probe DNA of *E*. *coli* O157:H7 was immobilized on three separate self-assembled layers of a TiO_2_ nanoparticle-DNA biosensor. The sensor was stored at room temperature inside a dry cabinet after the immobilization process.

**Fig 6:**
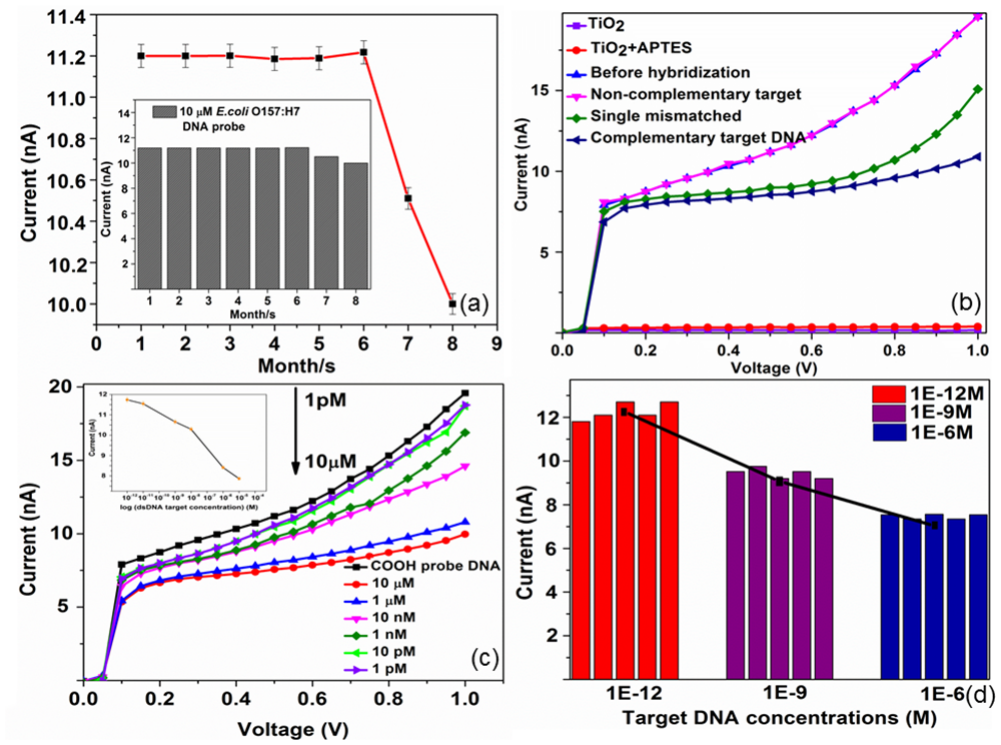
Responses on the sensing surface. (a) Current response of the 10 μM probe of *E*. *coli* O157:H7 ssDNA concentration measured over 8 months. Inset shows the corresponding histogram of the peak current values on a sensor for 8 months.(b) Current responses of different target DNA hybridized on the 10 μM probe DNA. These results proved the ability of the fabricated device to differentiate the positive and negative controls. (c)Current to voltage curves of the complemented target DNA concentrations. Inset shows the current values of different target DNA concentrations over the range 1E-12, 10E-12, 1E-9, 10E-9, 1E-6 and 10E-6 M. (d) Average current measurements of three different targeted DNA concentrations, 1 pM, 1 nM and 1 μM. The corresponding histogram shows the current values after 5 hybridization cycles.

The magnitude of the current response was monitoredfor 8 months periodically. The result shown in Fig 6a indicates that the developed biosensor retained activity for at least 6 months with an average current value of 11.2 nA. After this duration, the probe DNA coated on the sensor surface decreased gradually to 10.6 and 10 nA in the following 2 months of storage due to the stacking interaction of base pairs. Naturally, G/C base pairs have a stronger stacking interaction than A/T base pairs[35]. Thus, the A/T base pairs tend to be less stable even at low temperature with prolonged period. Moreover, the hydrogen bond between the bases would have to break as would up to two hydrogen bonds to water molecules when exposed to room temperature with high humidity. The relative standard deviation (RSD) calculated was 4.6%, and retains 99% of its original response current within 6 months.

### Specificity of the *E*. *coli* O157:H7 DNA Biosensor

In fabricating a DNA sensor, it is particularly important to self-assemble a monolayer to introduce a contact layer with oligomer DNA. Under atmospheric conditions, TiO_2_ metal oxide was terminated by the hydroxyl group (-OH),which allows the attachment of molecules via a condensation reaction. Therefore, APTES was used for a silanization process. The –OH groups were hydrolysed and formed siloxane bonds (Si-O-Si) with APTES. Fig 6b shows the basic curves of current to voltage of the TiO_2_ nanoparticle-based biosensor. At 0.5V, the current for TiO_2_ nanoparticles is 0.17 nA, and it slightly changes to 0.35 nA when APTES was simply dropped on the fabricated sensor. After immobilization process, the current value abruptly increases to 11.2 nA, according to the increase in surface charge density from the negatively charged DNA backbone. This method drastically reduces the solution effect; which is the noise from various chemicals as this method only uses DI water in DNA dilution.

After that, the device selectivity was further examined by hybridizing 3 different targets separately. There is a significant decrease in the current signal to 8.5 nA when a positive control was adoptedbecause of the ion blockage when it forms double-stranded DNA (ds-DNA). No significant change in signal was observed when the non-complementary target was exposed to the probe, showing the high selectivity of the constructed DNA biosensor for the hybridization detection. A 1-μM single base mismatched DNA sequence was adopted, and the current signal was 5.3% of that for perfectly matched DNA of the same concentration. This was further confirmed that the TiO_2_ nanoparticle-based IDEs could be used as an effective indicator to distinguish between inordinately mismatched oligonucleotides.

### Detection Limits of the *E*. *coli* O157:H7 TiO_2_ nanoparticle-based IDEs

Maximizing the immobilization process of the DNA sensor’s surface is the most crucial step. Thus, 10 μM carboxyl-probe DNA was introduced on the surface of the TiO_2_-based aluminium IDEs to evaluate the efficiency of different target DNA concentrations. Six *E*. *coli* O157:H7 DNA concentrations, 1 pM, 10 pM, 1 nM, 10 nM, 1 μM and 10 μM, were prepared by serial dilution in deionized distilled water. A small sample volume of 2.5 μl was takenfrom each concentration for picoammeter-sensing potential to evaluate the detection range by calculation of current.Fig 6c shows the measured current of the fabricated TiO_2_ biosensor taken at different target *E*.*coli* O157:H7 DNA concentrations with the help of the Keithley6487. The current nanoampere (nA) range is extremely low and can only be read by the Keithley6487 picoammeter. The current of the DNA biosensor decreases as the ssDNA target is hybridized due to the excessive negative chargethat comes from the DNA molecules. In other words, the resistance in the space charge region increases; thus, it resists the current to flow to the DNA sensor. The TiO_2_ biosensor itself has very low current,0.17 nA at 0.5V. This is one of the requirements to build a quantitativelow-volume DNA sensor as it would not damage the sensitive DNA.

The results show that the concentration of bacteria and output current were highly correlated. The output currentswere inversely proportional to the *E*. *coli* O157:H7 DNA concentration, mainly because the electrostatic interaction between the ssDNA probe and its complement target DNA affects the electrical properties and electron-transfer kinetics [36] of the TiO_2_ nanoparticles. The output currents of the ssDNA target were decreased further from the ssDNA probe as the target concentrations were increased. The peak current formed a linear relationship with the concentration of target over the range from 1 μM to 1 pM with the current value at 10.6 nA to 8 nA. There are binding modes between TiO_2_ nanoparticles and ds-DNA that are attributed to the intercalative binding [13]. At target DNA concentrations ≤ 1 pA, the current was less sensitive towards the 10 μM probe DNA, indicating small changes incharge transfer of ssDNA target towards the DNA biosensor, which is in good agreement with Li et al. [4]. Compared to many current research approaches that use electrochemical methodssuch as method proposed by Niu et al.[13], our picoammeter ismore sensitive; a detection limit of 10 fM was identified. Moreover, each detection consumes less than 30 minutes to obtain asteady result.

### Determination duplex formation using DNA from lysed *E*. *coli* O157:H7

**Fig 7:**
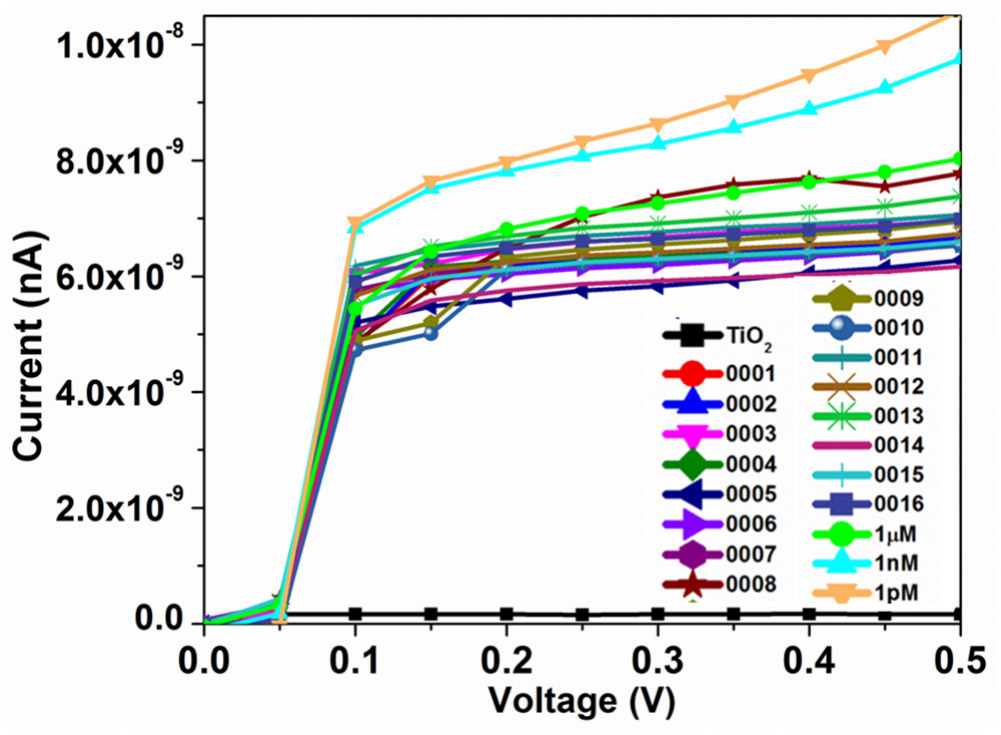
Current responses of different target DNA hybridized on the 10 μM probe DNA. The target DNA was from the lysed *E*. *coli* O157:H7. Different concentrations of target DNA were tested.

Samples were extracted and denatured by heating the solution in an incubator at 90°C for 2 min. The samples were frozen in an ice water bath for 1 min to obtain the sample solutions containing ssDNA sequence. Electrical changes after hybridization were recorded after dropping 2.5 μl of ssDNA sample solution on the surface of immobilized TiO_2_ nanoparticles—based interdigitated electrodes and shown in Fig 7. The current signals were decreased with increasing concentration of E.coli O157:H7 DNA shows the successful hybridization process with the formation of dsDNA on the electrode. Further, we could attain similar sensitivity as shown with synthetic DNA. This proved that the TiO_2_ nanoparticles—based interdigitated electrodes could effectively detect the lysed sample of E. coli O157:H7 without any amplification of DNA.

### Reproducibility and Repeatability of DNA Biosensor

The reproducibility of the DNA sensor for repeated detection of the *E*. *coli* O157:H7 targeted DNA was also investigated. The hybridization of 1 μM single-stranded targeted DNAand regeneration processes wererepeatedthree timeson fabricated DNA biosensors, and the output currents were observed. Regeneration of the TiO_2_nanoparticle-based IDEs to remove *E*.*coli* O157:H7 target DNA wasperformed by dropping 0.1M of NaOH liquid denaturation at room temperature for 6 min with a very small volume, 2.5 μl. The output currents of the denatured dsDNA oligonucleotides for three devices were monitored to be backed exactly to the ssDNA probe curve. This simple approach was selected to minimize the interface contact between liquid and electrode, which has the possibility to oxidize this biosensor and change the reading of the extremely small output current. The results show that the single-stranded target DNA of *E*. *coli* O157:H7 was completely detached from the carboxyl-modified probe without causing any damage to the DNA biosensors. NaOH was selected as the medium for dehybridization instead of NaCl [37, 38]because the use of NaCl solution tends to leave a salt precipitate on top of the device, even though it had been rinsed with DIwater. These resultsshow that this biosensor could retain 93% of its original response current after threecycles of regeneration, hybridization, and dehybridization. The relative standard deviation (RSD) of *E*. *coli* O157:H7 was calculated to be 1.6%, showing high regeneration and reproducibility of the developed TiO_2_ nanoparticle-based biosensor.

Next, the repeatability of the three separateDNA sensorswas investigated by measuring three different target concentrations (1 μM, 1 nM, 1pM) of single-stranded *E*. *coli* O157:H7 targeted DNA. These tests were conducted using the same procedures as described in the previous paragraph on separate sensors. The output currents of three different samples for each of the single-strand target DNA concentrations were measured five consecutive times at intervals of 24h as in Fig 6d. The sensors were stored at room temperature inside a dry cabinet before their first use. The RSD of 1 μM, 1 nM and 1pM targets were calculated to be 1.6%, 3.0% and 2.89%, respectively, for five repetitive measurements. These results indicated acceptable precision and fabrication reproducibility of the picoammeter-biosensor. The biosensor possessed 92%, 82% and 95.2%, of its original output current response for 1 μM, 1 nM and 1 pM targets, respectively. The present study demonstrated a rapid and ultra-high selectivity DNA biosensor that does not need real time PCR amplification or tissue culture steps to increase the *E*. *coli* O157:H7 DNA concentration. Further, the performance of sensor is shown here is significant and comparable/better than other sensing strategies currently available [39–45].

## Conclusions

In this work, we exploited a picoammeter to develop a biosensor platform that uses titanium dioxide nanoparticle-based interdigitated electrodes for the detection of *E*. *coli* O157:H7 DNA hybridization. For the detection of *E*. *coli* O157:H7 via TiO_2_ nanoparticle-based IDEs target DNAs from both commercial source and lysed *E*. *coli* O157:H7 were tested against probe sequence. The major difference of this picoammeter-biosensor from the conventional electrochemical method is that it isthe simplest method of biosensing because no chemicals are needed aside from DIwater. It is a novel technique that can be commercialized for real sample detection. The responsivity of this biosensor was thoroughly investigated by the simple current-to-voltage translation of a picoammeter. It is shown that this picoammeter-DNA biosensor was able to detect as low as 1.0 x 10^-13^M of *E*.*coli* O157:H7 ssDNA within a 30-minute, with high specificity and reproducibility.
